# Synthesis of castor oil/PEG as textile softener

**DOI:** 10.1038/s41598-024-56917-2

**Published:** 2024-03-26

**Authors:** H. M. Fahmy, A. Amr

**Affiliations:** Textile Research and Technology Institute, National Research Centre, Dokki, 12622 Giza Egypt

**Keywords:** Castor oil, Polyethylene glycol, Hybrid emulsion, Cotton fabric, Textile softener, Materials science, Nanoscience and technology, Chemistry, Organic chemistry, Polymer chemistry, Surface chemistry, Chemical synthesis

## Abstract

New castor oil/polyethylene glycol (CAO/PEG) hybrids were synthesized by reacting of CAO with PEG 300, 600, 1000, 2000 or 4000, in presence of ammonium per sulfate (APS) as an initiator. The optimum conditions to synthesis such hybrids are: PEG/CAO weight ratio, 35%; APS/PEG weight ratio, 15%; reaction temperature, 80 °C; and reaction time, 60 min. Only the hybrids based on PEG 1000 and 2000 formed oil in water stable emulsions. Treating cotton fabric samples with easy care finishing formulation containing 40 g/L of the synthesized hybrids emulsions results in an enhancement in softness, tensile strength, whiteness index, and stiffness along with a reduction in nitrogen content, wrinkle recovery angle, and wettability properties of treated fabric, compared to that sample finished in absence of that emulsions. The chemical structure of the synthesized CAO/PEG1000 hybrid was confirmed via the FTIR and ^1^HNMRanalysis whereas the TEM analysis showed that the particles size of that hybrid emulsion is in the range of 27–105 nm. Moreover, such hybrid emulsion treated fabric surface was characterized via SEM and EDX analysis. Furthermore, treating dyed samples with the nominated hybrid emulsion improves the color strength of that samples but keeps the washing fastness, wet rubbing fastness as well as alkaline perspiration fastness of the dyed/finished samples unchanged. The wet rubbing fastness and alkaline perspiration fastness of all the dyed/finished samples were enhanced while the light fastness of such samples decreased.

## Introduction

Recently, extensive research has been made to enhance the textile comfortability and functionality such as the anti-bacterial protection, UV blocking, self-cleaning, easy care, water repellency and softness taking in consideration both the environment and consumer concerns^1–4^. Developing of multifunctional textile products can be achieved by innovating new functional finishes as well as replacing the harsh chemical processes with promising nano-, bio-and/orplasma technological processes to widen the potential applicability, enhance competitiveness, as well as minimize energy, water, and chemicals consumption^5–7^.

A textile softener is a chemical which that modifies the fabric hand causing to be of pleasing touch. Soft finishes achieve textiles with soft hand, smoothness, flexibility and better drape properties. In addition, softeners enhance tearing strength, improve abrasion resistance, as well as reduce sewing thread breakage. Due to these merits, soft finishes are nearly included in every finishing formulation of textiles. The softener is a two-ended molecule, one end being hydrophilic and the other a lyophilic long hydrocarbon chain. According to the molecule ionic nature, softeners are widely classified into anionic, cationic and nonionic^8–10^. The nonionic softeners, such as ethoxylates, esters, polyethylenes, silicones, and waxes, have the merits of excellent compatibility with the other ingredients of the finishing bath, efficient fiber lubrication as well as the high resistance to yellowing. Because of their low affinity to textile they are applied to textiles by the padding method. Moreover, they have limited durability to washing or dry cleaning as they do not form covalent bonds with textile fibers^8–11^.

**Figure 1:**
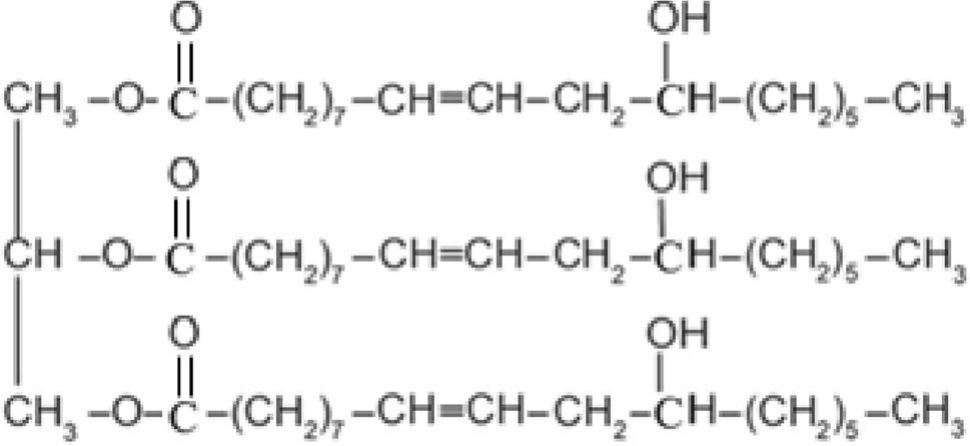
Chemical structure of castor oil^3^.

Castor oil (Fig. 1), among the vegetable oils, is naturally oil composed mainly of the ricinoleic acid that contains both a hydroxyl group and double bond rendering castor oil to be a starting material as a polyol in various chemical reactions and industrial applications^3,12–15^. Emulsions of Castor oil are widely used in the cosmetics, pharmaceutical, and food industry^16^. Gums like Arabic gum and Terminalia gum can be used in castor oil emulsification^17,18^. Previous study by Zhang et al. reported that the best emulsifier formula to achieve castor oil stable emulsion is a mixture of polyoxyethylene sorbitan monostearate and sorbitan monooleate^19^. Sulfated castor oil is used as an anionic textile softener namely Turkey red oil^9^. Castor oil, coconut Oil, and vegetable Ghee were used to synthesize eco-friendly textile softeners^20^. Two new textile softeners were synthesized by treating a mixture of castor oil and poly (*N*-vinyl-2-pyrrolidone) thermally at 150 °C^21^ or in presence of ammonium per sulfate at 80 °C^22^.

The present work was under taken to establish the proper conditions to emulsify castor oil using polyethylene glycol in presence of ammonium per sulfate as an initiator.

## Experimental

### Materials

Mill scoured and bleached cotton fabric of weave structure, weight of 128 g/m^2^ and count (Ne) of 40/1 was supplied by Misr Spinning and Weaving Co., Mahalla El—Kobra, Egypt. Pure castor oil (CAO) supplied by the oils extraction unit of the National Research Centre, Dokki, Cairo, Egypt, was used. Durapret®LF (DMDHEU), low formaldehyde resin of conc. 70%, Biotex Company, was used as a crosslinker for easy care finishing of cotton fabric. Polyethylene glycol (PEG) of molecular weight 300, 600, 1000, 2000, and 4000 Dalton, supplied by Alpha chemiKa, was used. Silver nitrate, ammonium per sulfate (APS) and ammonium sulfate of laboratory-grade chemical were used. The following commercial direct and reactive dyes were used: C.I. Reactive Black 5, C.I. Reactive Yellow 2, and C.I. Reactive Red 15.

### Methods

#### Synthesis of CAO/PEG hybrid

The CAO/PEG hybrid was synthesized as follows: a mixture of specific weight of CAO and PEG of different weight ratios to CAO (0–20%) was stirred at 70 °C in 150 ml stoppered glass bottle in an air circulated oven. To that mixture, APS concentrated solution, 582 g/l^23^, in which the APS is in weight ratio to the PEG (0–30%) was added and the reaction was then left under stirring to proceed at different temperatures (70–90 °C)for different periods of time (15–90 min).

#### Preparation of the CAO/PEG hybrid emulsion

At the reaction end, specific volume of hot distilled water at 70 °C is added to the reaction medium followed by homogenizing the reaction products, using a strong homogenizer, for 3 min to form homogeneous oil in water emulsion.

#### Reaction product separation

Components of the CAO/PEG hybrid that prepared at optimum reaction conditions was separated as follows: 14 g of the hybrid were vigorously stirred with 300 ml n-hexane in a 500 ml beaker to dissolve the castor oil that does not contain any PEG species in its chemical structure leaving a precipitate which was then washed again five times with 70 ml n-hexane to completely purify that precipitate. After that, 70 ml of acetone were added to such precipitate with stirring to dissolve castor oil grafted with PEG species and sulfate groups leaving different oxidized species as a small weight precipitate (≈ 0.37 g) that was filtered while the acetone was evaporated from the remnant. Visually, only the castor oil grafted with PEG species formed oil in water emulsion. On the other hand, percent of the castor oil grafted with PEG species was estimated using the following equation^24^:

$$\% {\text{G}} = \left[ {\text{PEG grafted CAO}} \right]{/}\left[ {{\text{CAO}}} \right] \times {1}00\%$$

#### Preparation of silver nanoparticles

Silver nano-particles (Ag-NPs) were prepared using tri-sodium citrate as a reductant as reported elsewhere^25^.

#### Fabric treatment

Cotton fabric samples of 30 × 30 cm^2^ were padded twice in easy care finishing bathes containing DMDHEU as a crosslinker and ammonium persulfate as a catalyst in presence or absence 40 g/l of the prepared CAO/PEGhybrid emulsion. The padded samples were squeezed to a wet pick up of 100%, dried at 100 °C/3 min in Wenner Mathis AGCH-8155 oven and then cured at 150 °C/3 min. The finished fabrics were then washed with distilled water at 50 °C for 10 min, thoroughly rinsed and finally dried for testing.

#### Dyeing of cotton fabric

The cotton fabric samples were dyed with the aforementioned reactive dyes as follows: 1% of any of such reactive dyes (owf) were dissolved in distilled water at 40 °C and then 30 g/l Na_2_SO_4_ were added to the dyeing bath. After that, the fabric samples were added to the dyeing bath using a material to liquor ratio 1:50 and the bath was kept at 40 °C for 30 min. The dyeing bath temperature was then raised to 60 °C in case of C.I. Reactive Black 5 or to 80 °C for the C.I. Reactive Red 15 and C.I. Reactive yellow 2 followed that by an addition of 15 g/l sodium carbonate and then the dyeing process was left at the mentioned above temperatures for 60 min. After that, the dyed samples were soaped using 3% nonionic detergent, rinsed, and dried at room temperature.

### Analysis and test methods

- Fabric weight was assessed according to ATSM (D 3776-79).
- The nitrogen content (%N) was evaluated according to Kjeldahl method^26^.
- The wrinkle recovery angle of treated fabric samples (WRA) was determined according to ASTM method D-1296-98.
- Wettability (W) of the finished fabric sample was assessed according to AATCC Test method 39-1980.
- Whiteness Index (WI), color strength (K/S), and colorimetric data (L*, a*, b* and ∆E) of treated fabric samples were evaluated using Ultra Scan PRO from Hunter Lab. The color difference (∆E) between the dyed and dyed/finished fabric samples was evaluated by considering the unfinished dyed sample as a standard sample.
- Surface roughness (SMD) was measured using Kawabata evaluation system, Surface tester KES-FB4-A, Kato Tech Co., LTD, Japan.
- Tensile strength (TS) was tested in the warp direction according to ASTM procedure D-2256-98.
- Stiffness (S) was determined in the warp direction according to ASTM Test Method D 1388-96 using Jika (Toyaseiki) apparatus.
- The fastness properties of the dyed cotton fabric samples to washing (WF), rubbing (RF), perspiration (PF), and light were assessed due to ISO test methods: 105-C06 (2010), 105-X12 (1987), 105-E04 (2008), and 105-B02(2013) respectively.
- The antibacterial activity was assessed using the bacterial count method as reported elsewhere^21^ against:
  - Gram-positive bacteria: Staphylococcus aureus (SA).
  - Gram-negative bacteria: Escherichia coli (EC).
- Durability to washing was assessed by subjecting the finished fabric to 10and 20 washing cycles according to AATCC test method 61-1994.
- Fourier-transform infrared spectroscopy (FTIR) was carried out using FT/IR-4700 FTIR Spectrometer from JASCO.
- The ^1^H NMR spectra were obtained in deuterated chloroform (CDCl_3_) using 300 MHz Varian Mercury Plus NMR Spectrometer.
- Particle size distribution of the prepared silver nano-particles was evaluated using Zeta sizernano series (Nano ZS, ZEN 3600), Marven Co., UK.
- The morphology and particles size of the hybrid emulsion was obtained by transmission electron microscope (TEM) using a JEOL, JEM 2100 F electron microscope at 200 kV.
- Scanning electron microscope (SEM) images of the treated and untreated fabric samples were obtained using SEM Model Quanta 250 FEG (Field Emission Gun) attached with EDX Unit (Energy Dispersive X-ray Analyses), with accelerating voltage 30 kV, magnification 14× up to 1,000,000 and resolution for Gun, FEI company, Netherlands.

## Results and discussion

### Tentative mechanism

It is anticipated that addition of APS aqueous solution to a thermostated chemical reaction containing a mixture of polyethylene glycol and castor oil present in plenty compared to PEG would cause the following reactions^27–29^:

- Generation of free radicals
  1 $${\text{S}}_{2} {\text{O}}_{8}^{2 - } \to 2{\text{SO}}_{4}^{ - \cdot }$$
  2 $${\text{SO}}_{4}^{ - \cdot } + {\text{H}}_{2} {\text{O}} \to {\text{HSO}}_{4}^{ - } + {}^{ \cdot }{\text{OH}}$$
  3 $${\text{PEG-OH}} + {\text{R}}^{ \cdot } \to \mathop {{\text{PEG-O}}^{ \cdot } }\limits_{{({\text{PEG}}\;{\text{macroradicals}})}} + {\text{RH}}$$
  where R^⋅^ is the free radicals species (SO^⋅^_4_^−^ and/or HO^⋅^) generated by the decomposition of ammonium per sulfate in aqueous medium.
- Reaction of castor oil via double bonds^28,29^:45
- Reaction of castor oil via hydroxyl groups^27,29^:6
- Reaction of castor oil via both the double bonds and hydroxyl groups^27,29^:7

Thus, the aforementioned reaction medium includes a mixture of un-reacted castor oil, castor oil grafted with PEG species and/or sulfate groups, as well as different oxidized species; all in state of entanglement. However, the grafted castor oil acts as a self-emulsifier for the remaining unreacted castor oil since castor oil is present in the reaction medium in plenty with respect to PEG. For simplicity, this mixture will be referred as CAO/PEG hybrid. The major factors affecting CAO/PEG hybrid formation will be studied. Results obtained along with appropriate discussion follow.

### Factors affecting CAO/PEG hybrid formation

#### Initiator concentration

Table 1 shows the effect of APS/PEG weight ratio on CAO/PEG hybrid emulsion state. It is clear that, increasing APS/PEG weight ratio to 15% is accompanied by a gradual improvement in the hybrid emulsion state which could associated with increasing of the free radicals number initiating the formation of CAO-g-PEG species as an emulsifier responsible for emulsification of the remaining unreacted CAO, since the PEG/CAO weight ratio is 35%. The further increasing in that ratio, within the range studied, has no effect on the emulsion state, suggesting the rapid rate of termination due to increasing of the APS concentration^1,2,28^.

**Table 1:** Effect of APS/PEG weight ratio on the CAO/PEG hybrid emulsion state.

APS/PEG weight ratio (%)	Emulsion state
0	No emulsion
5	Emulsion with tinny oil drops
15	Stable emulsion
25	Stable emulsion

PEG MW, 1000 Dalton; PEG/CAO weight ratio, 35%; reaction temperature, 80 °C; reaction time, 60 min.

#### Reaction temperature

Table 2 illustrates the impact of reaction temperature on the CAO/PEG hybrid emulsion state. As is evident, raising the reaction temperature from 60 to 80 °C significantly improves hybrid emulsion state, the point that reflects the temperature favorable effect on the initiate or decomposition, i.e. increasing of the free radicals species, as well as increasing of the reactants molecules mobility, the matter that certainly enhances formation of the CAO-g-PEG species as emulsifier and consequently state of the hybrid emulsion^1,2,28^. Moreover, the further increase in reaction temperature, i.e. beyond 80 and up to 90 °C, has no effect on the hybrid emulsion state. It seems that at higher temperatures, the high rate of initiation is outweighed by a faster rate of termination^1,2,28^.

**Table 2:** Effect of reaction temperature on the CAO/PEG hybrid emulsion state.

Temperature (°C)	Emulsion state
60	Emulsion with large oil drops
70	Emulsion with tinny oil drops
80	Stable emulsion
90	Stable emulsion

PEG MW, 1000 Dalton; PEG/CAO weight ratio, 35%; APS/PEG weight ratio, 15%; reaction time, 60 min.

#### Reaction time

Table 3 shows the CAO/PEG hybrid emulsion stateas a functionin the reaction time. It is obvious that increasing of the reaction time from 15 to 60 results in a gradual improvement in the hybrids emulsion state as a result for increasing of APS extent of decomposition that in turn enhances the hybrid formation as well as upgrades its emulsion state. Prolonging the reaction time does not affect state of the hybrid emulsion which can be interpreted in terms of the depletion in the PEG amount and/or APS concentration^30–32^.

**Table 3:** Effect of reaction time on the CAO/PEG hybrid emulsion state.

Time (min)	Emulsion state
15	No emulsion
30	Emulsion with large oil drops
45	Emulsion with tinny oil drops
60	Stable emulsion
90	Stable emulsion

PEG MW, 1000 Dalton; PEG/CAO weight ratio, 35%; APS/PEG weight ratio, 15%; reaction temperature, 80 °C.

#### PEG/CAO weight ratio

Table 4 depicts the effect of the PEG/CAO weight ratio on emulsion state of the formed hybrid. It is well seen that increasing of that ratio from 0 to 35% in the reaction medium gives rise to a progressive promotion in the hybrid emulsion state. This may be a direct consequence for increasing of the CAO-g-PEG species formation in the reaction medium in addition to the role of the unreacted PEG in dispersing of the formed hybrids^2,3,28,33^. The further increasing in that ratio, i.e. at 45%, does not affect the hybrid emulsion state.

**Table 4:** Effect of PEG/CAO weight ratio on the CAO/PEG hybrid emulsion state.

PEG/CAO weight ratio (%)	Emulsion state
0	No emulsion
10	Emulsion with large oil drops
25	Emulsion with tinny oil drops
35	Stable emulsion
45	Stable emulsion

PEG MW, 1000 Dalton; APS/PEG weight ratio, 15%; reaction temperature, 80 °C; reaction time, 60 min.

#### PEG molecular weight

Table 5 shows the effect of PEG molecular weight on emulsion state of the prepared hybrids. It is clear that increasing of PEG molecular weight from 300 to 1000 Da is accompanied with a gradual promotion in emulsion state of the formed hybrid. Moreover, using of the PEG of the molecular weight 2000 Da in preparation of the CAO/PEG hybrids keeps stability of the hybrid emulsion. Unfortunately, PEG 4000 impairs state of the formed hybrid emulsion which may be related to increasing of the reaction medium viscosity that hinders the molecular collisions of the reactants and/or decreasing of the hydroxyl groups number by increasing the PEG molecular weight, the matter that indeed reduces the extent of formation of CAO-g-PEG species^3,33^.

**Table 5:** Effect of PEG molecular weight on the CAO/PEG hybrid emulsion state.

PEG molecular weight (Dalton)	Emulsion state
300	Emulsion with large oil drops
600	Emulsion with tinny oil drops
1000	Stable emulsion
2000	Stable emulsion
4000	Emulsion with tinny oil drops

PEG/CAO weight ratio, 35%; APS/PEG weight ratio, 15%; reaction temperature, 80 °C; reaction time, 60 min.

### Inclusion of the CAO/PEG hybrids emulsionsin easy-care finishing of cotton fabric

#### The performance properties of the CAO/PEG hybrids emulsions treated fabric

Table 6 shows the effect of incorporating of CAO/PEG1000 or CAO/PEG2000 hybrid emulsion in the easy care finishing formulations of cotton fabric on performance properties of that fabric. It is obvious that inclusion of either of such hybrids emulsions in the finishing bath leads to an enhancement in nitrogen content, wrinkle recovery angle, stiffness, and softness along with a reduction in tensile strength, whiteness index, and wettability properties of treated fabric, compared to the untreated fabric sample. The matter that can be associated with the fixation of such hybrids ingredients onto/within the treated fabric structure^3,21,22^ as represented by Eq. (8). Moreover, during the reaction of binding of that hybrids ingredients to the cotton fabric (Eq. 8), other reactions may be also occurred that are crosslinking of the cotton cellulose (Eq. 9) and/or crosslinking of the hybrid ingredients (Eq. 10) forming a deposit onto/within the finished fabric resulting in extra softness effect to the finished fabric surface.8910

**Table 6:** Some performance properties of the CAO/PEG hybrids emulsions treated fabric.

Type of the hybrid	%N	WRA (w + f)^O^	TS (kg)	W (S)	WI	S (mg)	SMD (µm)
Untreated	0	156 (± 3.1)	45 (± 3.1)	1 (± 0.16)	71.32 (± 4.5)	605.2 (± 6.7)	1.816 (± 0.16)
CAO/PEG1000 hybrid	0.2435 (± 0.005)	244 (± 3.1)	32 (± 2.5)	31 (± 1.9)	67.1 (± 4.1)	854.4 (± 5.7)	1.479 (± 0.16)
CO/PEG2000 hybrid	0.2154 (± 0.003)	234 (± 3.5)	34 (± 3.5)	49 (± 2.2)	67.4 (± 3.7)	925.6 (± 6.1)	1.529 (± 0.21)

The hybrid synthesis conditions: PEG MW, 1000 Da, PEG/CAO weight ratio, 35%; APS/PEG weight ratio, 15%; reaction temperature, 80 °C; reaction time, 60 min. Finishing conditions: The hybrids emulsions conc., 40 g/l; [DMDHEU], 60 g/l; [(NH_4_)_2_SO_4_], 2 g/l; wet pick up, 100%; drying, 100 °C/3 min; curing, 160 °C/3 min.

Table 6 illustrates also that replacing the CAO/PEG1000 with CAO/PEG2000 in the above mentioned finishing bath leads to an enhancement in extents of tensile strength, whiteness index, and stiffness along with a reduction in nitrogen content, resiliency, wettability and softness properties of the treated fabric. It seems that increasing of the PEG molecular weight from 1000 to 2000 Da renders the CAO/PEG2000 hybrid emulsion particles to be larger than that of the CAO/PEG2000 hybrid emulsion^34–38^ resulting in a rougher treated fabric surface^38^ as well as alteration in extents of the above mentioned properties.

#### The antibacterial properties of the CAO/PEG hybrids emulsions treated fabric

Finishing conditions: [Hybrids emulsions], 40 g/l; [DMDHEU], 60 g/l; [(NH_4_)_2_SO_4_], 4 g/l; [Ag-NPs], 2 g/l; wet pick up, 100%; drying, 100 °C/3 min; curing, 160 °C/3 min. G+ ve: St. aurous; G− ve: *E. coli*. Values in parentheses indicate retained antibacterial properties of treated fabric after 10 and 20 laundering cycles.

Table 7 shows the antibacterial properties of cotton fabric samples treated with different finishing formulations containing the aforementioned hybrids emulsions in absence or presence of silver nanoparticles (Ag-NPs). It is obvious that: (1) incorporating of such hybrids emulsions, regardless of the hybrid type, brings about an enhancement in the antibacterial activities of treated fabric against both the G− ve (*E. coli*) and G+ ve (*S. auereus*) bacteria reflecting the antibacterial properties of castor oil^39,40^, (2) the fabric sample treated with the CAO/PEG1000 hybrid emulsion has higher antibacterial activities than that treated with the CAO/PEG2000 hybrid emulsion; a point that reflects the antibacterial properties of PEG 1000^41^, (3) inclusion of Ag-NPs in the easy care finishing bathes containing any of the above mentioned hybrids emulsions results in upgrading of the antibacterial properties of treated fabric. The matter that can be related to a generation of silver ions, in the presence of moisture, that attach to the bacterial DNA causing its inactivation according to Eq. (11) and/or generating of oxygen radicals that destroys the bacteria molecular structure according to Eq. (12)^21^, and (4) the antibacterial properties of both the hybrids emulsions treated fabric care durable for 20 washing cycles, suggesting the role of DMDHEU as a cross linker in binding of such hybrids ingredients to the cotton cellulose structure (Eq. 6).

11 $${\text{O}}_{{2({\text{aq}})}} + 4{\text{H}}_{3} {\text{O}}^{ + } + 4{\text{Ag}}_{{(s)}} \to 4{\text{Ag}}^{ + } _{{({\text{aq}})}} + 6{\text{H}}_{2} {\text{O}}$$

12 $${\text{H}}_{2} {\text{O}} + (1{\text{/}}2){\text{O}}_{2} \xrightarrow{{{\text{Ag}}^{ + } }}{\text{H}}_{2} {\text{O}}_{2} \to {\text{H}}_{2} {\text{O}} + ({\text{O}})$$

**Table 7:** The antibacterial properties of the CAO/PEG hybrids emulsions treated fabric.

Finishing formulation	Reduction (%)	
	G+ve	G−ve
Untreated	0	0
CAO/PEG1000 hybrid emulsion	94.2 ± 3.5 (91.4 ± 2.2) (85.5 ± 3.8)	92.1 ± 2.1 (89.4 ± 2.9) (82.9 ± 3.9)
CAO/PEG2000 hybrid emulsion	92.1 ± 2.1 (89.2 ± 2.9) (82.1 ± 3.2)	90.3 ± 3.6 (89.1 ± 3.2) (80.3 ± 4.1)
CAO/PEG1000 hybrid emulsion/Ag-NPs	99.5 ± 2.5 (95.1 ± 4) (88.7 ± 3.5)	97.4 ± 3.6 (93.6 ± 2.3) (86.2 ± 4.5)
CAO/PEG2000 hybrid emulsion/Ag-NPs	98.1 ± 3.3 (94.3 ± 3.7) (85.1 ± 4.2)	95.7 ± 3.5 (92.3 ± 3.4) (83.4 ± 4.1)

**Figure 2:**
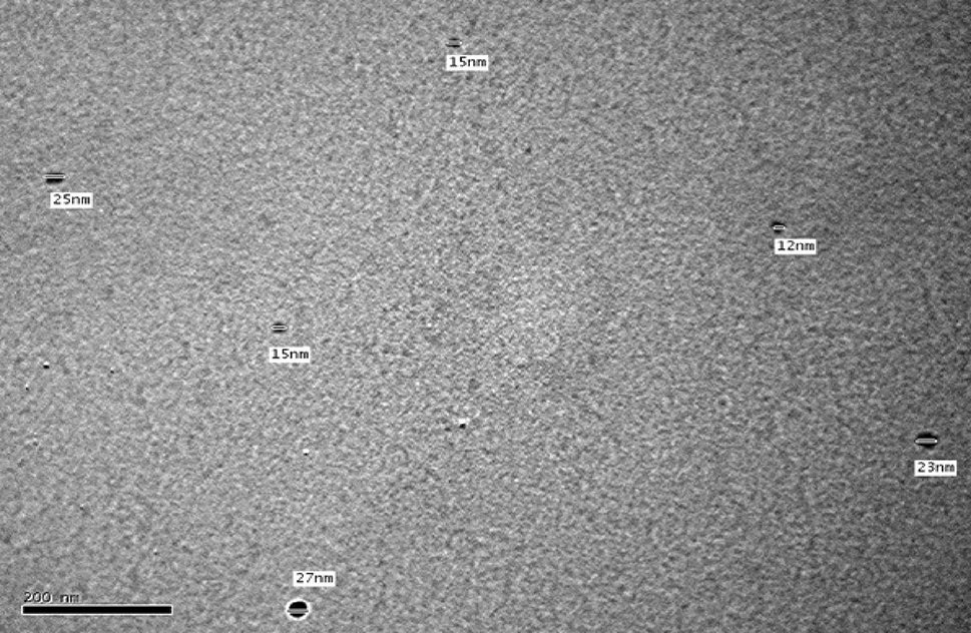
TEM image of the prepared Ag-NPs.

**Figure 3:**
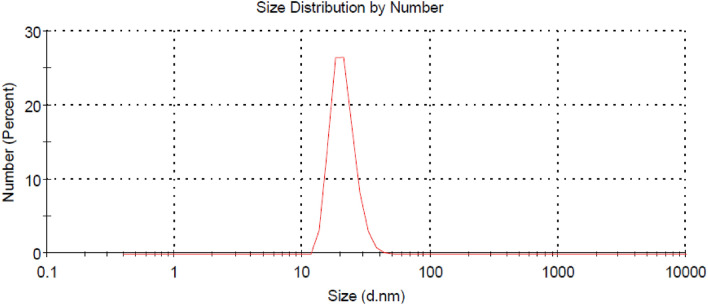
Particle size distribution of the prepared Ag-NPs.

On the other hand, Fig. 2 shows the TEM image of the prepared Ag-NPs Whereas Fig. 3 shows the particle size distribution of such Ag-NPs. It is clear from Fig. 2 that size of the prepared Ag-NPs ranges from 15 to 27 nm whereas Fig. 3 confirms that the meandiameter of such particles is 21.04 nm.

### Characterization of CAO/PEG1000 hybrid

**Figure 4:**
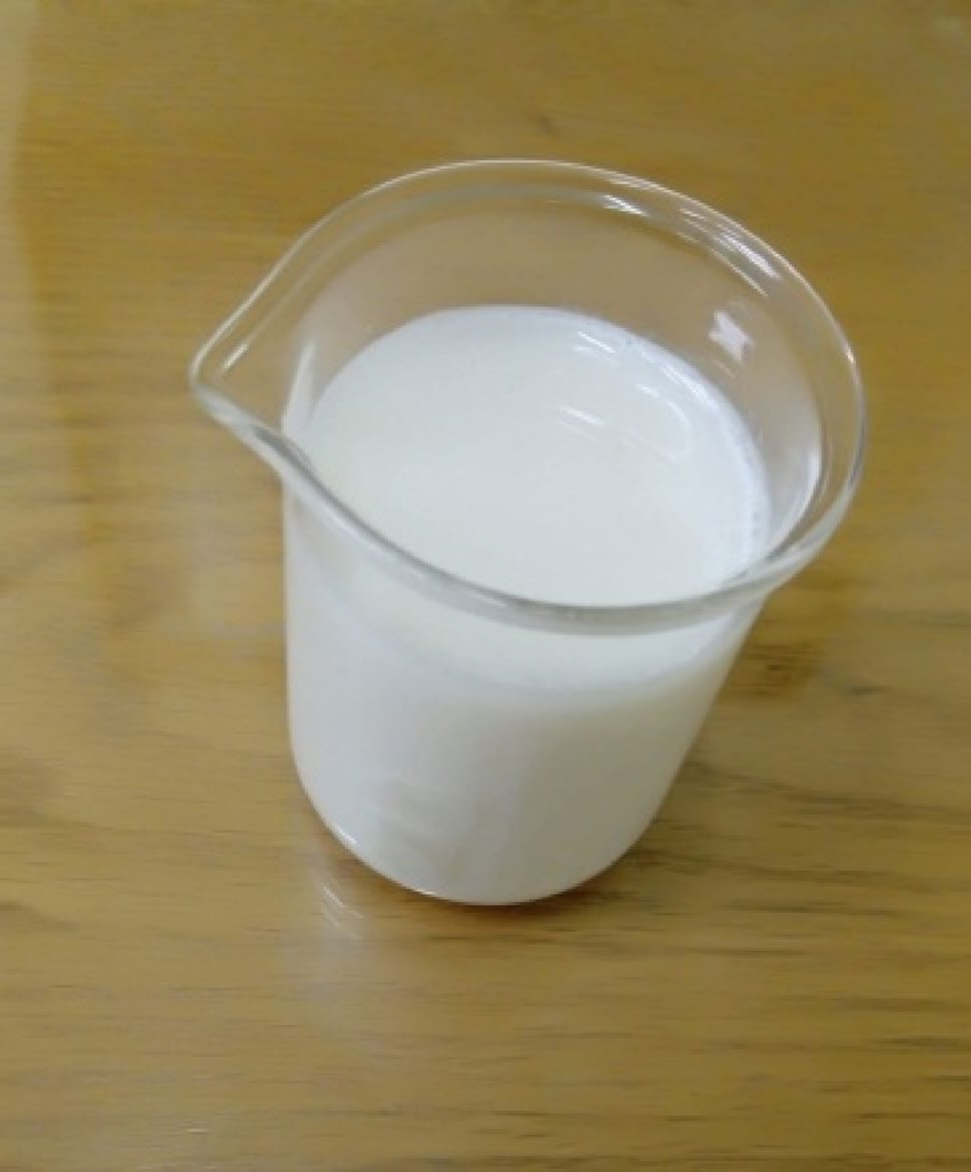
Visual appearance of the prepared CAO/PEG1000 hybrid emulsion.

Due to the performance as well as antibacterial properties of the CAO/PEG1000 hybrid emulsion treated fabric, it is selected to be characterized via FTIR and ^1^HNMRanalysis. Moreover, such hybrid emulsion particle size was examined via TEM analysis whereas its treated fabric was characterized via SEM and EDX analysis. In addition, Fig. 4 shows the visual appearance of the CAO/PEG1000 hybrid emulsion. On the other hand, the percent grafting of castor oil with PEG 1000 was calculated using the following equation^24^:

$$\% {\text{G}} = \left[ {\text{PEG grafted CAO}} \right]{/}\left[ {{\text{CAO}}} \right] \times {1}00\%$$

It was found that percent grafting was equal to 65.1% by considering weight of the PEG grafted castor oil equals 6.51 g and the original weight of castor oil is 10 g.

#### FTIR analysis

**Figure 5:**
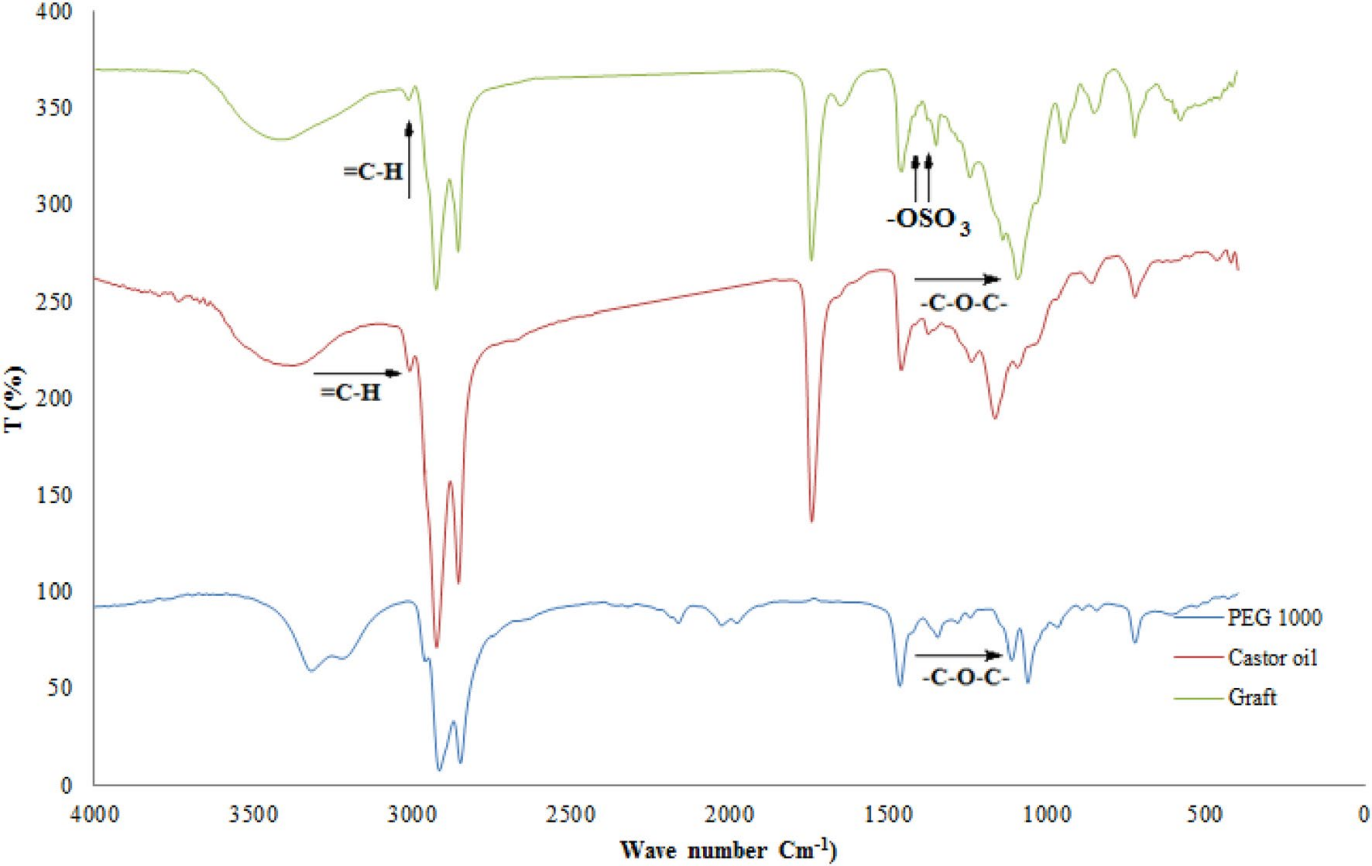
FTIR of castor oil, PEG 1000, and PEG grafted castor oil.

The FTIR spectra of castor oil, PEG 1000, and grafted castor oil are represented by Fig. 5. The spectrum of castor oil includes a peak at 3408 cm^−1^ corresponding to –OH stretching vibration, a peak at 2975 cm^−1^ corresponding to C–H (=C–H) stretching vibration, peaks at 2916 and 2848 cm^−1^ corresponding to CH_2_ asymmetrical and symmetrical respectively, a peak at 1734 cm^−1^ corresponding to C=O stretching of the ester group, a peak at 1624 cm^−1^ corresponding to the double bond of castor oil, and a peak at 941 cm^−1^ corresponding to –C=C– (trans) bending out of plane^13–15,42^. Moreover, the PEG 1000 spectrum includes peaks at 3301 cm^−1^ corresponding to –OH stretching, 2890 and 2840 cm^−1^ corresponding to aliphatic CH_2_ stretching vibration, 1452 cm^−1^ is due to binding vibration of CH_2_, and 1101 cm^−1^ corresponding to C–O–C stretching^3^.

On the other hand, the grafted castor oil spectrum includes some peaks resemble that of castor oil and PEG1000 such as a peak at 3401 cm^−1^ corresponding to –OH stretching of castor oil and PEG 1000, a peak at 3037 cm^−1^ corresponding to C–H (=C–H) stretching vibration of castor oil but with a lower intensity than that of the castor oil which may indicate opening of some double bonds of the castor oil during the hybrid formation, peaks at 2929 and 2858 cm^−1^ corresponding to aliphatic CH_2_ stretching vibration of castor oil and PEG 1000, a peak at 1745 cm^−1^ corresponding to C=O stretching of the ester group of castor oil, and a strong peak at 1101 cm^−1^ corresponding to –C–O–C– group but with a higher intensity than that of the PEG 1000 which confirms grafting of the castor oil viaits double bonds^27,29^. In addition, new peaks were appeared in the grafted castor oil spectrum at 1415 and 1380 cm^−1^ corresponding to sulfate groups which indicates replacement of some of –OH groups of castor oil with sulfate groups^27,29,43^.

#### ^*1*^*HNMR analysis*

**Figure 6:**
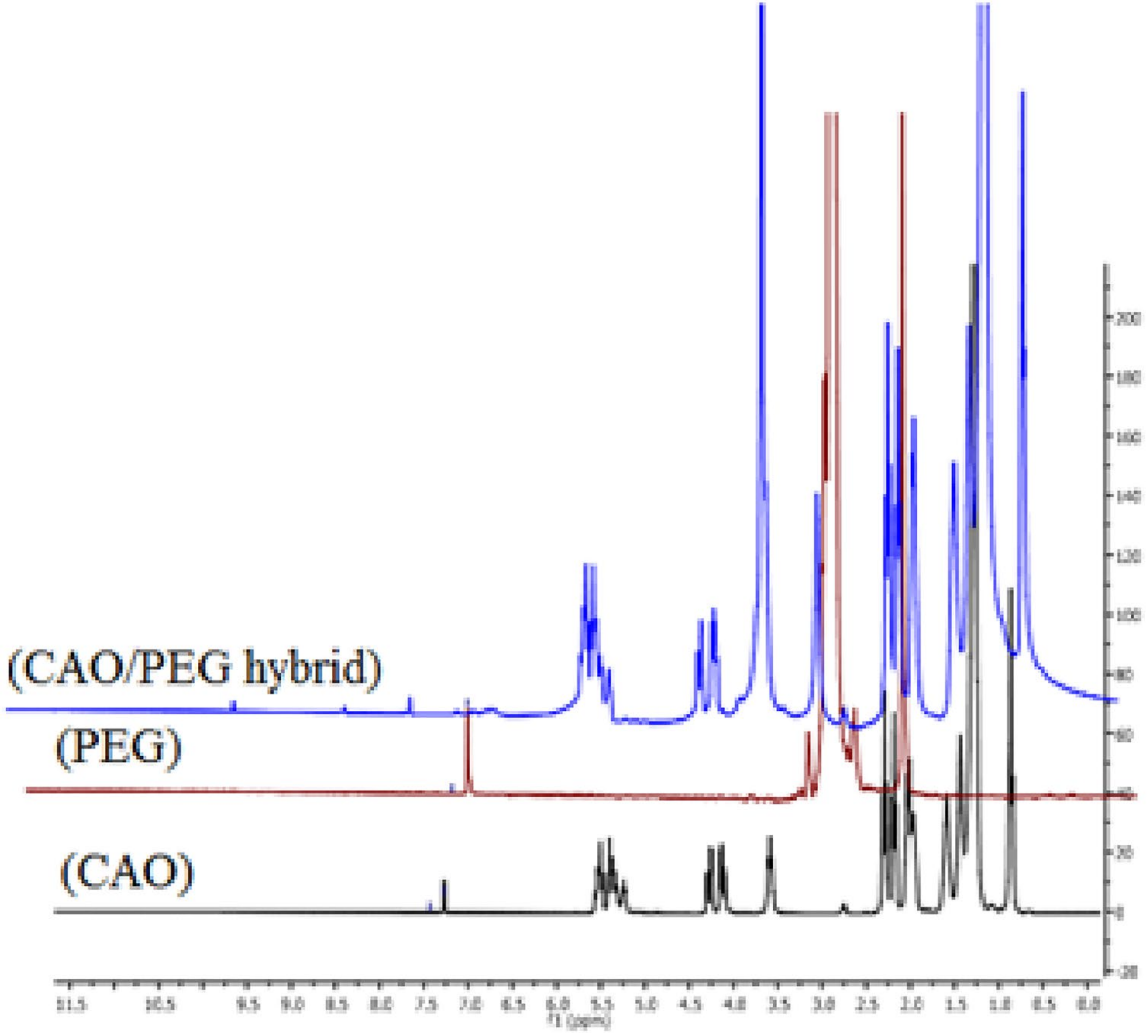
^1^HNMR of CAO/PEG1000 hybrid, castor oil and PEG1000.

Figure 6 shows the ^1^H NMR of CAO/PEG1000 hybrid, castor oil and PEG1000. The following peaks were observed: 0.5–1 (s, Me), 1.0–1.5 (m, CH_2_), 1.75–2.0 (m, CH_2_ beside double bond), 2.0–2.5 (m, CH_2_ beside ketone and double bond), 3.0 (s, OH), 3.5 (s, OCH_2_), 4.0–4.5 (dd, olefinic-H), 5–5.5 (m, olefinic-H). By comparing the ^1^H NMR spectra of castor oil, polyethylene glycol, and the CAO/PEG1000 hybrid, it was noticed that there is an increasing in the integration of –CH_2_, –OCH_2_ and –OH groups indicating grafting of castor oil with polyethylene glycol.

#### TEM image

**Figure 7:**
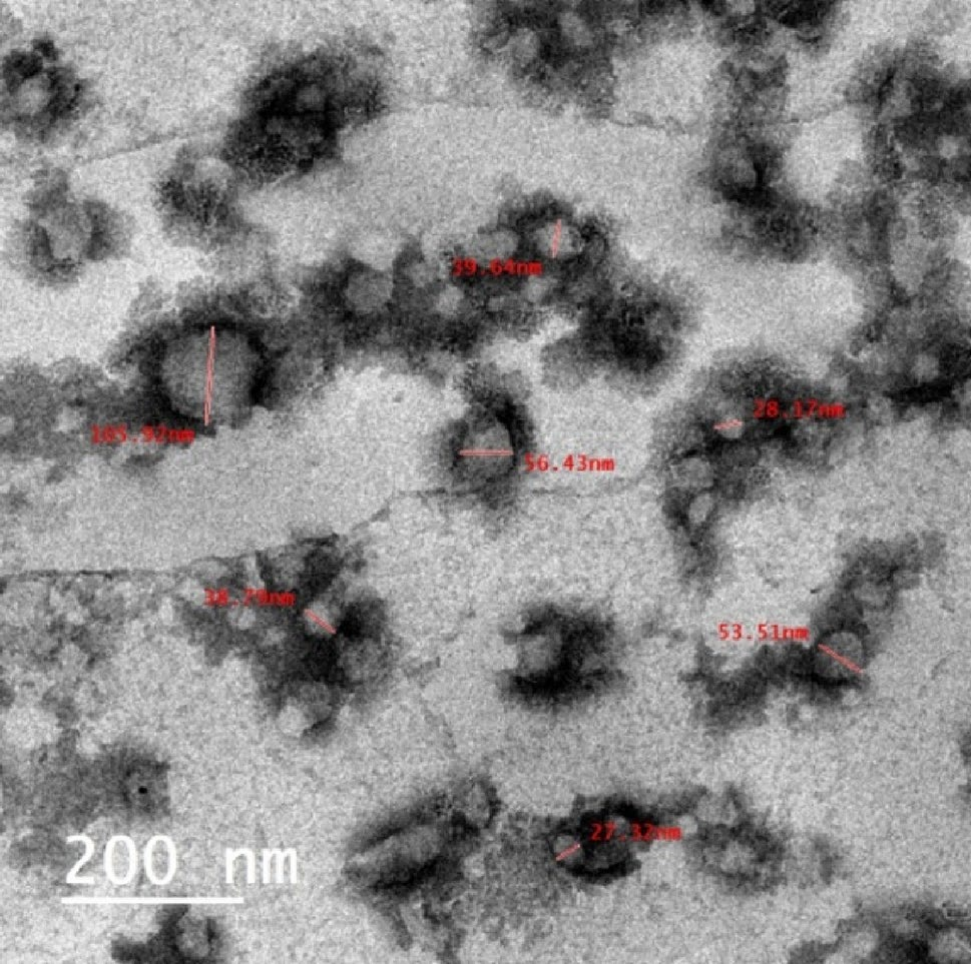
TEM image of the CAO/PEG1000 hybrid emulsion.

The particles size of the CAO/PEG1000 hybrid emulsion was characterized via TEM analysis and represented by Fig. 7. It is observed that such emulsion particles size is in the range of 27–105 nm.

#### SEM and EDX images

**Figure 8:**
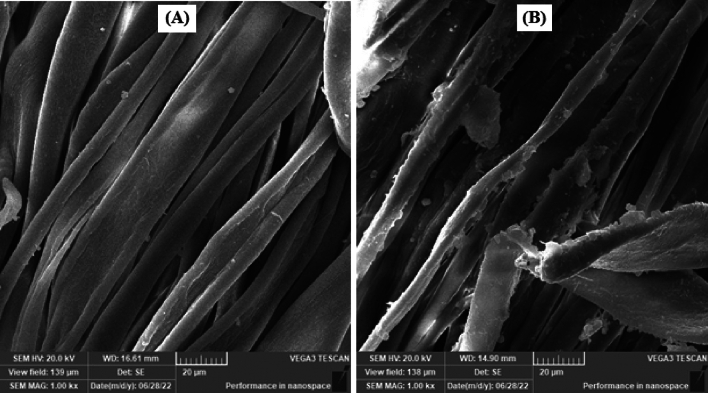
SEM images of (**A**) untreated fabric and (**B**) CAO/PEG1000 hybrid emulsion treated fabric.

**Figure 9:**
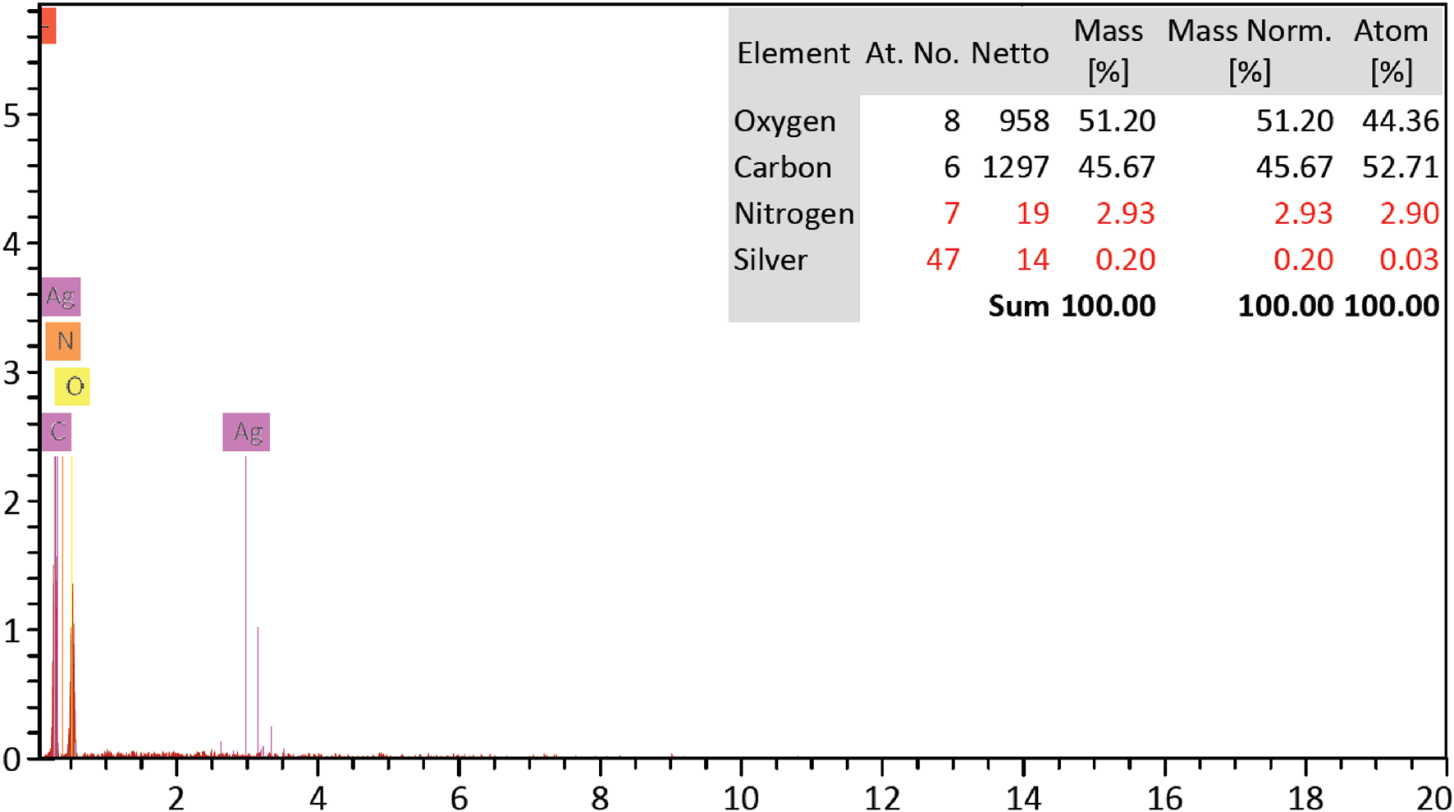
EDX image of the CAO/PEG1000 hybrid emulsion containing Ag-NPs treated fabric.

Figure 8 represents the SEM images of untreated cotton fabric and treated fabric with easy care finishing bath containing the prepared CAO/PEG1000 hybrid emulsion. It is well seen that the treated fabric surface has a deposited film. On the other hand, Fig. 9 shows the EDX analysis of the Ag-NPs loaded CAO/PEG1000 hybrid emulsion treated fabric which confirms the presence of Ag-NPs onto the finished fabric structure with Ag-content of 0.20% (w/w).

### The wavelength, coloration and fastness properties of the dyed and dyed/finished cotton fabric

According to the higher performance as well as antibacterial properties of the CAO/PEG1000 hybrid emulsion, compared to the CAO/PEG2000 hybrid emulsion, cotton fabric samples were dyed with three reactive dyes and then treated with easy care finishing formulation containing that hybrid emulsion to illustrate the impact of that finishing on wavelength, coloration and fastness properties of the dyed samples. Given below the results along with an appropriate discussion.

#### The wavelength and coloration properties

**Figure 10:**
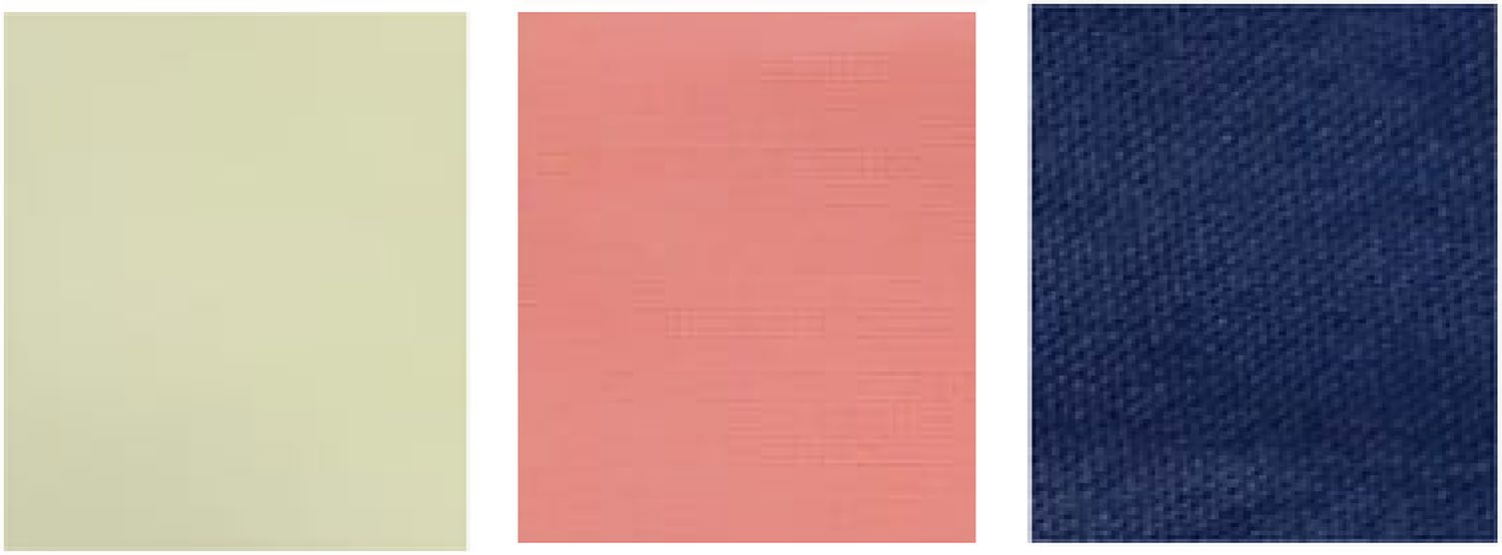
Reactive Yellow 2, Reactive Red 15, and Reactive Black 5 dyed samples finished with the CAO/PEG1000 hybrid emulsion.

The wavelength (λ_max_), color strength (K/S), as well as colorimetric data (L*, a*, b* and ∆E) magnitudes of the dyed cotton fabric samples before and after their treating with easy care finishing bath containing the CAO/PEG1000 hybrid emulsion are represented by Table 8. It is obvious that the un-finished dyed samples have different wavelengths, color strengths and colorimetric data reflecting the variation in functionality, molecular size, affinity, and mode of interaction of the nominated dyes^44^. Moreover, finishing of the dyed samples with the CAO/PEG1000 hybrid emulsion, regardless of reactive dye chemical structure, results in an increasing in K/S as well as a noticeable change in ∆E^45^ of the dyed/finished fabric samples, the matter that can be associated with the color-deepening effect of the CAO/PEG1000 hybrid emulsion as a textile softener^21^. Figure 10 shows the Reactive Yellow 2, Reactive Red 15, and Reactive Black 5 dyed samples treated with the CAO/PEG1000 hybrid emulsion.

**Table 8:** The effect of the dye type on wavelength, color strength, and coloration properties of the dyed and dyed/finished cotton fabric.

Dye type	λ_max_	K/S		L*		a*		b*		∆E
		BF	AF	BF	AF	BF	AF	BF	AF	
Reactive Black 5	610	7.34	7.56	39.75	38.95	− 7.82	− 8.02	− 17.62	− 16.43	1.45
Reactive Yellow 2	405	0.44	0.50	82.95	81.69	− 4.60	− 4.18	14.17	14.49	1.37
Reactive Red 15	515	1.66	1.69	63.97	63.21	33.56	32.78	5.36	5.53	1.11

BF: before finishing; AF: after finishing.

#### The fastness properties

The fastness properties of the dyed and dyed/finished fabric samples are represented by Table 9. Table 9 signifies that: (1) treating the dyed samples with nominated hybrid emulsion has no effect on washing fastness, dry rubbing fastness as well as acidic perspiration fastness of such samples, (2) the wet rubbing fastness and alkaline perspiration fastness of all the dyed/finished samples were enhanced, and (3) the light fastness of all the dyed/finished samples was decreased. The alteration in fastness properties of the dyed/finished fabric samples reflects the effect of deposition of the synthesized CAO/PEG hybrid ingredients onto the dyed samples structure.

**Table 9:** The fastness properties of the dyed and dyed/finished fabric samples.

Dye type	WF						RF				PF												Light	
											Acidic						Alkaline							
	St		St*		Alt		Dry		Wet		St		St*		Alt		St		St*		Alt			
	BF	AF	BF	AF	BF	AF	BF	AF	BF	AF	BF	AF	BF	AF	BF	AF	BF	AF	BF	AF	BF	AF	BF	AF
Reactive Black 5	4	4	4	4	4	4	4	4	4	4–5	4	4	4	4	4	4	4	4	4	4	4	4–5	5	4–5
Reactive Yellow 2	4–5	4–5	4–5	4–5	4–5	4–5	4–5	4–5	4	4–5	4–5	4–5	4–5	4–5	4–5	4–5	4	4–5	4	4–5	4	4–5	6	5–6
Reactive Red 15	4–5	4–5	4–5	4–5	4–5	4–5	4–5	4–5	4	4–5	4–5	4–5	4–5	4–5	4–5	4–5	4	4–5	4	4–5	4	4–5	5	4–5

Alt: alteration in color; St: staining on cotton; St: staining on wool; BF: before finishing; AF: after finishing.

### CAO/PEG hybrids emulsion storage stability

Upon storing any of the aforementioned prepared hybrids emulsions for 1 month, tinny oil drops appear after the third day that by shaking a homogeneous emulsion is formed again. After one month, the emulsions separated into two layers; a turbid solution in the bottom and a thicker emulsion above it. The bottom layer height is about one third of the upper layer. However, upon shaking any of such emulsions, a homogeneous emulsion is reformed again.

## Conclusions

- New CAO/PEG hybrids were synthesized by reacting CAO with PEG 1000 or PEG 2000 Da, in presence of APS as an initiator.
- The optimum conditions to synthesis such hybrids are: PEG/CAO weight ratio, 35%; APS/PEG weight ratio, 15%; reaction temperature, 80 °C; and reaction time, 60 min.
- The prepared hybrids stable emulsions were applied as textile softeners during easy care finishing of cotton fabric.
- Upon treating cotton fabric samples with easy care finishing formulations containing 40 g/l of such hybrids emulsions, the performance properties of that fabric samples such as tensile strength, whiteness index, stiffness, and softness were enhanced but the nitrogen content, wrinkle recovery angle, and wettability were reduced, compared to the control fabric sample.
- Increasing the PEG molecular weight results in a reduction in extents of the nitrogen content, wrinkle recovery angle, wettability and softness along with an enhancement in tensile strength, whiteness index, and stiffness properties of treated fabric.
- The chemical structure of the synthesized CAO/PEG1000 hybrid was confirmed by the FTIR and ^1^H NMR analysis whereas the TEM analysis showed that the particles size of that hybrid emulsion in the range of 27–105 nm.
- The CAO/PEG1000 hybrid emulsion treated fabric surface was characterized via SEM and EDX analysis.
- Treating dyed samples with nominated hybrid emulsion improves the color strength of such samples but keeps the washing fastness, wet rubbing fastness as well as alkaline perspiration fastness of the dyed/finished samples unchanged.
- The wet rubbing fastness and alkaline perspiration fastness of all the dyed/finished samples were promoted while the light fastness of such samples decreased.

## Data Availability

The datasets used and/or analyzed during the current study are available from the corresponding author on reasonable request.
